# Diazepam induced sleep spindle increase correlates with cognitive recovery in a child with epileptic encephalopathy

**DOI:** 10.1186/s12883-021-02376-5

**Published:** 2021-09-14

**Authors:** S. M. Stoyell, B. S. Baxter, J. McLaren, H. Kwon, D. M. Chinappen, L. Ostrowski, L. Zhu, J. A. Grieco, M. A. Kramer, A. K. Morgan, B. C. Emerton, D. S. Manoach, C. J. Chu

**Affiliations:** 1 Department of Neurology, Massachusetts General Hospital, 175 Cambridge St, Suite 340, Boston, MA 02114 USA; 2 Department of Psychiatry, Massachusetts General Hospital, Boston, MA 02114 USA; 3 Harvard Medical School, Boston, MA 02115 USA; 4 Massachusetts General Hospital, Psychology Assessment Center, Boston, MA 02114 USA; 5 Department of Mathematics and Statistics, Boston University, Boston, MA 02115 USA

**Keywords:** CSWS, Continuous spike and wave of sleep with encephalopathy, Epileptic encephalopathy, High-dose diazepam, Case report

## Abstract

**Background:**

Continuous spike and wave of sleep with encephalopathy (CSWS) is a rare and severe developmental electroclinical epileptic encephalopathy characterized by seizures, abundant sleep activated interictal epileptiform discharges, and cognitive regression or deceleration of expected cognitive growth. The cause of the cognitive symptoms is unknown, and efforts to link epileptiform activity to cognitive function have been unrevealing. Converging lines of evidence implicate thalamocortical circuits in these disorders. Sleep spindles are generated and propagated by the same thalamocortical circuits that can generate spikes and, in healthy sleep, support memory consolidation. As such, sleep spindle deficits may provide a physiologically relevant mechanistic biomarker for cognitive dysfunction in epileptic encephalopathies.

**Case presentation:**

We describe the longitudinal course of a child with CSWS with initial cognitive regression followed by dramatic cognitive improvement after treatment. Using validated automated detection algorithms, we analyzed electroencephalograms for epileptiform discharges and sleep spindles alongside contemporaneous neuropsychological evaluations over the course of the patient’s disease. We found that sleep spindles increased dramatically with high-dose diazepam treatment, corresponding with marked improvements in cognitive performance. We also found that the sleep spindle rate was anticorrelated to spike rate, consistent with a competitively shared underlying thalamocortical circuitry.

**Conclusions:**

Epileptic encephalopathies are challenging electroclinical syndromes characterized by combined seizures and a deceleration or regression in cognitive skills over childhood. This report identifies thalamocortical circuit dysfunction in a case of epileptic encephalopathy and motivates future investigations of sleep spindles as a biomarker of cognitive function and a potential therapeutic target in this challenging disease.

## Background

Continuous spike and wave of sleep with encephalopathy (CSWS) is a rare and challenging epileptic encephalopathy characterized by seizures and abundant interictal spike (IIS) activity during non-rapid eye movement (NREM) sleep, concurrent with cognitive regression or failure to develop as expected relative to same-aged peers [1, 2]. Within the epileptic encephalopathies, presentations range from abundant focal IISs associated with subtle cognitive deficits in Rolandic epilepsy to focal, multifocal, or generalized IISs and dramatic cognitive regression in the syndrome of continuous spike and wave of sleep (CSWS) with encephalopathy [3–6]. Although studies have identified a genetic etiology in only a minority of cases, these reveal that patients with pathogenic variants in the same gene can present along a spectrum of disease severity [7], suggesting that a complex interplay between genes and endogenous and exogenous environmental variables mediate the clinical symptoms.

Converging evidence implicates dysfunction of thalamocortical circuits in CSWS and related epileptic encephalopathies. The sleep activated IIS activity implicates involvement of thalamocortical circuits that regulate sleep electrophysiology [8, 9]. Neonates with thalamic hemorrhages [10] and patients with other early thalamic injury [11] are at high risk of developing sleep activated IISs. Similarly, thalamic lesions are more common in children with CSWS compared to patients with developmental regression without CSWS [12] and are found to be lateralized to the affected side in cases of unilateral CSWS [13]. Focal white matter microstructural [14] and macrostructural [15] abnormalities have been observed in the thalamocortical white matter in children with Rolandic epilepsy, suggesting thalamocortical circuits can be focally disrupted in sleep activated epilepsy.

Efforts to link IISs and cognitive function in epileptic encephalopathies have not identified a consistent relationship. Surprisingly, in some cases, effective treatment correlates with increased IISs [16]. Sleep spindles - discrete bursts of 10–15 Hz oscillations during NREM sleep - are generated in the thalamus and linked to sleep dependent memory functions [17], but have scarcely been analyzed in sleep activated epilepsies. Sleep spindles originate in the thalamic reticular nucleus and are propagated in thalamocortical feedback circuits. Sleep spindles correlate with general measures of intelligence and overnight memory performance improvement and have been shown to be causally linked to sleep dependent memory consolidation in animal work [17–20].

Further supporting the potential role of sleep spindles in the pathophysiology of epileptic encephalopathies, IIS and spindles can be generated by the same thalamocortical circuits [21, 22]. Pharmacological manipulation can transform spindles into spike and wave activity in both slice preparations [23] and in vivo animal models [24]. Similar pathological oscillations can be found in genetic knockout models that modify connections within and to the reticular nucleus of the thalamus [25, 26]. Recently, we found that children with Rolandic epilepsy have focal spindle deficits in the same cortical regions with IIS, that spindle rate anticorrelates with IIS rate, and predicts cognitive symptoms even in the absence of a relationship between cognitive symptoms and spike rate [27]. These converging findings suggest that developmental sleep-activated epileptic encephalopathies are thalamocortical circuit disorders and that sleep spindles may provide an accessible and objective mechanistic biomarker for cognitive dysfunction.

In this case report, we apply advanced detection approaches to quantify IISs, sleep spindles, and cognitive function longitudinally in a child with CSWS with encephalopathy. We find that a marked increase in spindle rate coincided with high-dose diazepam treatment and a dramatic improvement in cognitive function. These observations demonstrate thalamocortical circuit dysfunction in a case of epileptic encephalopathy and motivate future investigations of sleep spindles as a biomarker of cognitive function and a potential therapeutic target in this challenging disease.

## Case presentation

### Clinical course

A 4.9 year old right-handed girl presented to the epilepsy clinic with a history of attention difficulties, mild hypotonia, and staring spells. A routine EEG found no electrographic correlate with her staring spells but she was incidentally found to have near-continuous IIS during NREM sleep. She underwent a detailed neuropsychological evaluation at age 5.4 years, which revealed cognitive delay. Based on these combined electro-clinical features, she was started on levetiracetam due to concerns that an epileptic encephalopathy may be contributing to her cognitive delay.

**Fig. 1:**
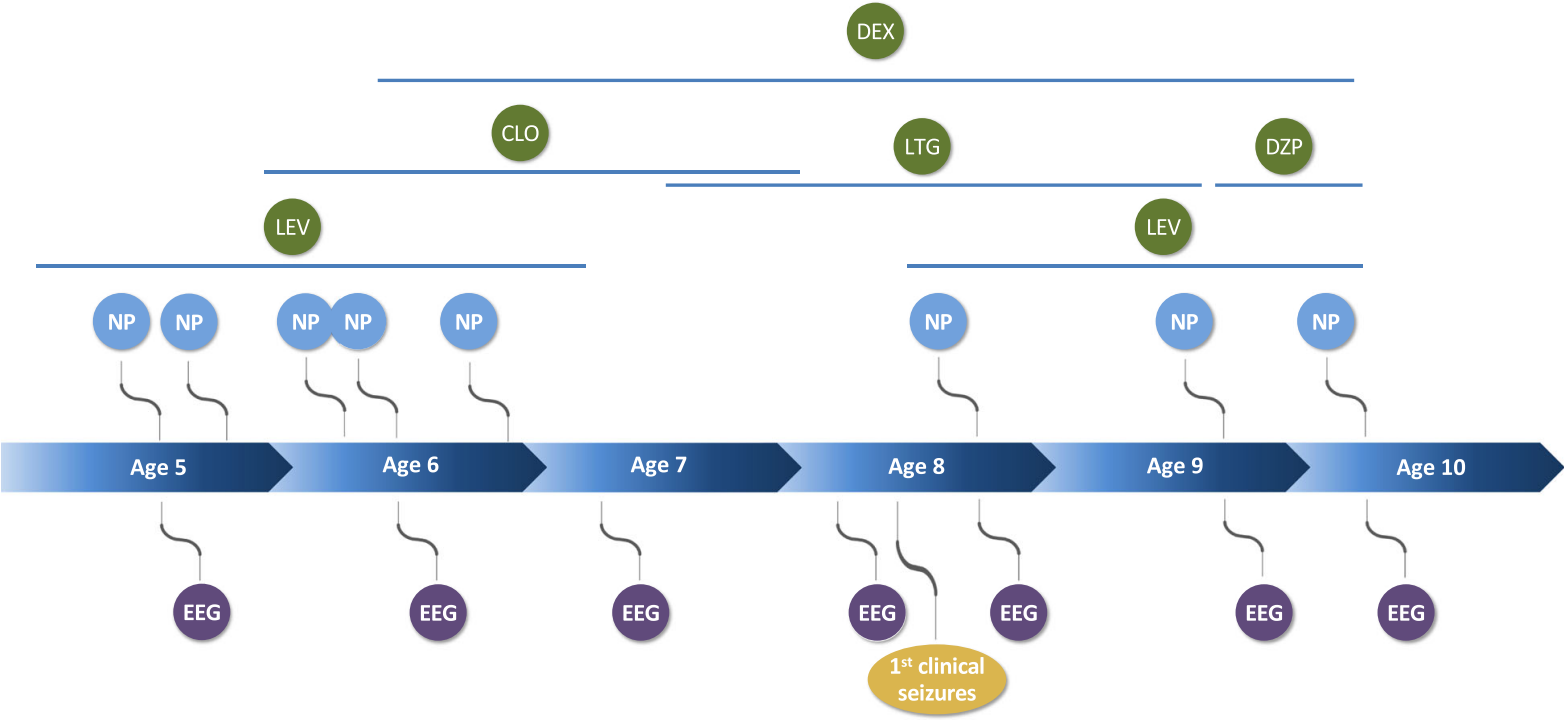
Longitudinal EEGs, neuropsychological (NP) evaluations, and medication changes over 6 years of follow-up. Neuroactive medications are indicated in green circles. LEV:Levetiracetam; CLO:Clobazam; LTG:Lamotrigine; DZP: Diazepam, DEX: Dexmethylphenidate

Over the next 6 years, the patient was followed closely with annual overnight EEGs (*n* = 7) and neuropsychological evaluations (*n* = 8), which occurred on the same day when possible (*n* = 5). She was treated with clobazam from age 5.8–8.0, lamotrigine from age 7.5–9.6, and levetiracetam from 4.9–7.0 and again from 8.4–10.0. There was no clear impact of any of these medications on her cognitive performance or EEG. Stimulant treatment with dexmethylphenidate was started for attention concerns at age 6.4 years. At the age of 8.4 years, she developed clinical seizures. The complete clinical time course is shown in Fig. 1.

**Fig. 2:**
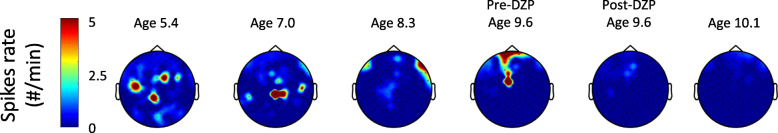
Longitudinal IIS rate during N2 sleep. Topographical map of the spike rate across ages shows a dramatic decrease on the night of high-dose diazepam treatment that was sustained with treatment. Spikes were detected with Persyst and were restricted to HD-EEG recordings

Neuropsychological testing at age 8.4 years identified relative and absolute regressions in multiple cognitive domains, especially relating to language. At 9.6 years, testing confirmed continued cognitive regressions, and in the setting of near-continuous IISs during NREM sleep, she was formally diagnosed with CSWS with encephalopathy. She was admitted for a 48-h EEG at this time and treated with high-dose diazepam (1 mg/kg) on hospital day two. Her IISs on EEG dramatically improved on the night of treatment and the patient and her family noted an immediate improvement in attention without any noted side effects (Fig. 2). She was discharged on hospital day three on diazepam (0.5 mg/kg) which was tapered over the next 4 months to 0.2 mg /kg. Repeat EEG at 10.1 years was normal and neuropsychological testing confirmed a marked improvement in cognitive performance.

### Neuropsychological findings

Between the ages of 5.4 and 10.1, the patient underwent eight clinical neuropsychological evaluations. The tests included age-based standardized scores of general and domain specific cognitive function. Age-appropriate neuropsychological measures were used for each evaluation. For assessments of verbal and language processing: Expressive One-Word Picture Vocabulary Test-4 (all ages), Wechsler Intelligence Scale for Children - 5th Edition (WISC-V) Verbal Comprehension Index (ages 6 and above), were evaluated. For assessments of auditory attention and working memory: WISC-V Digit Span (ages 6 and above), Wide Range Assessment of Memory and Learning - 2nd Edition (WRAML-2) Sentence Memory (ages 6 and above). For assessment of processing speed: Wechsler Preschool & Primary Scale of Intelligence – 4th Edition (WPPSI-IV) Bug Search (ages 5 and 6) and WISC-V Processing Speed Index (ages 6 and above) were evaluated. For assessments of motor performance: the Purdue Pegboard and Beery-Buktenica Developmental Test of Visual Motor Integration - 6th Edition (VMI-6) were evaluated (all ages). Select assessment of academic achievement included subtests from the Wechsler Individual Achievement Test - 3rd Edition (WIAT-III) Reading Comprehension and Math Problem Solving (ages 6 and above) were done with less frequency.

**Fig. 3:**
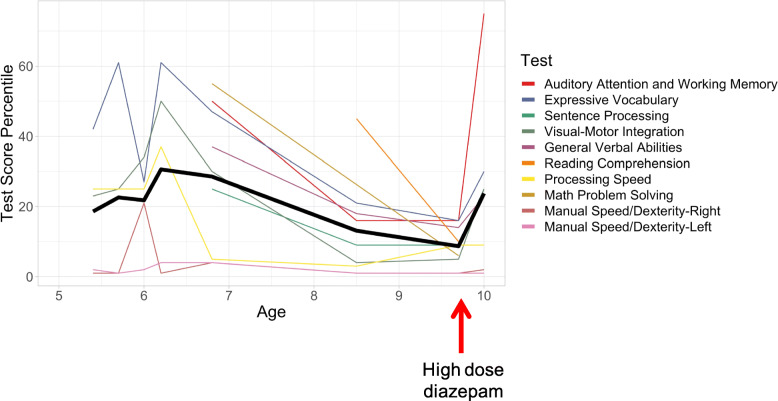
Course of neuropsychological testing scores. The thick black line is the mean across all cognitive scores shown. A consistent decline across cognitive domains is apparent from age 6.4 years to age 9.6 years after which improvements were observed following high-dose diazepam treatment

On initial presentation, the patient was noted to perform in the lower percentiles for age across some domains tested. Her initial performance vacillated across different domains from ages 5.4–6.2 years. From 6.2 to 9.6 years, frank regressions were evident in both age-corrected performance and raw scores across all domains, including in auditory attention and working memory, general verbal abilities, expressive vocabulary, sentence processing, reading comprehension, mathematics, processing speed, and visuomotor integration. Testing 4 months after high-dose diazepam treatment revealed improvements in all domains tested except motor dexterity and processing speed (Fig. 3).

### Electroencephalographic findings

Between the ages of 5.4 and 10.1, the patient underwent 7 overnight EEG recordings. For each EEG recording, the patient was admitted to the epilepsy monitoring unit and EEG data were collected with either low-density (21 EEG electrodes following the 10–20 system, *n* = 2) or high-density electrodes (128 electrodes, ANT-Neuro Waveguard, *n* = 5) using a Natus amplifier. Sampling frequency varied from 256 to 1024 Hz. All EEG electrodes were placed by registered EEG technicians and impedances were below 10 kΩ.

EEGs were manually reviewed, and sleep scored according to standard criteria [28]. Epochs with large movement artifacts were rejected, and channels contaminated by continuous artifact were manually identified. Of the 8 overnight EEG recordings analyzed, a mean of 289 min (range: 256–326) of N2 was recorded and mean of 270 min (range: 248–299) was analyzed after removing epochs containing arousals or artifactual activity. Among the 128 channel recordings, a mean of 4.1 bad channels were identified and interpolated from neighboring channels using fieldtrip [29] (range per recording 1–12). Among 21 channel recordings, a mean of 1.5 bad channels were identified and ignored (range per recording 1–2). Independent component analysis was used to remove EKG artifacts. Data were bandpass filtered (0.1–35 Hz) and referenced to the common average of all channels. Stage 2 NREM (N2) sleep epochs were concatenated and downsampled to 256 Hz for automated spindle and spike detection. Here, we focused on N2 because this is the sleep stage that has most consistently been correlated with sleep-dependent memory consolidation [30].

#### Sleep spindle activity over disease course

**Fig. 4:**
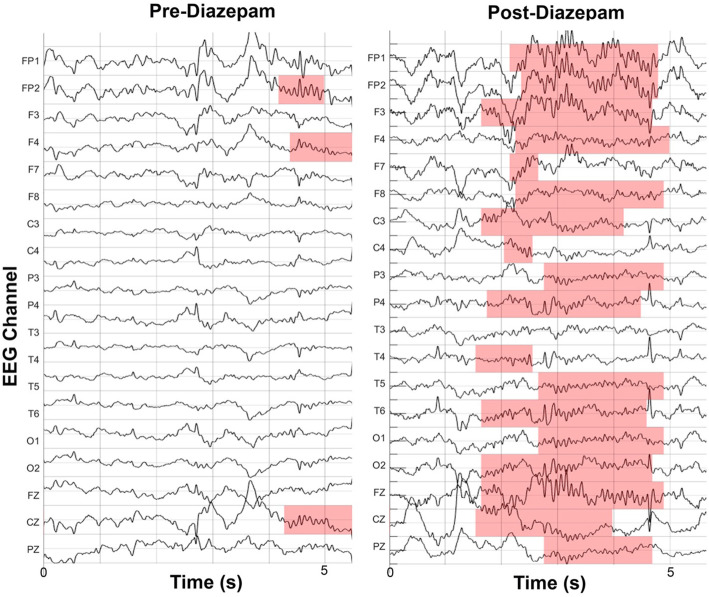
Example spindle detections at age 9.6 years pre- and post- high dose diazepam treatment. Data is referenced the common average and spindle detections at each channel are highlighted. For detection, spindles are required to have regular oscillations that stand out from the background activity in the sigma band (9–15 Hz), not have an increase in theta activity (4-8 Hz) which can occur with epileptiform spikes, and last at least 0.5 s in duration [27]

Sleep spindles were quantified using a latent state automated spindle detector validated to robustly detect spindles in patients with IISs [27]. This detector uses theta power (4–8 Hz), sigma power (9–15 Hz), and the Fano factor to reliably distinguish spindles from background activities, artifacts, and IIS, which can each cause spurious increases in sigma power calculations. The detector works on a 0.5 s sliding window and when the calculated probability of a spindle was greater than 95% in this window, a spindle was detected. Example spindle detections are shown in Fig. 4.

**Fig. 5:**
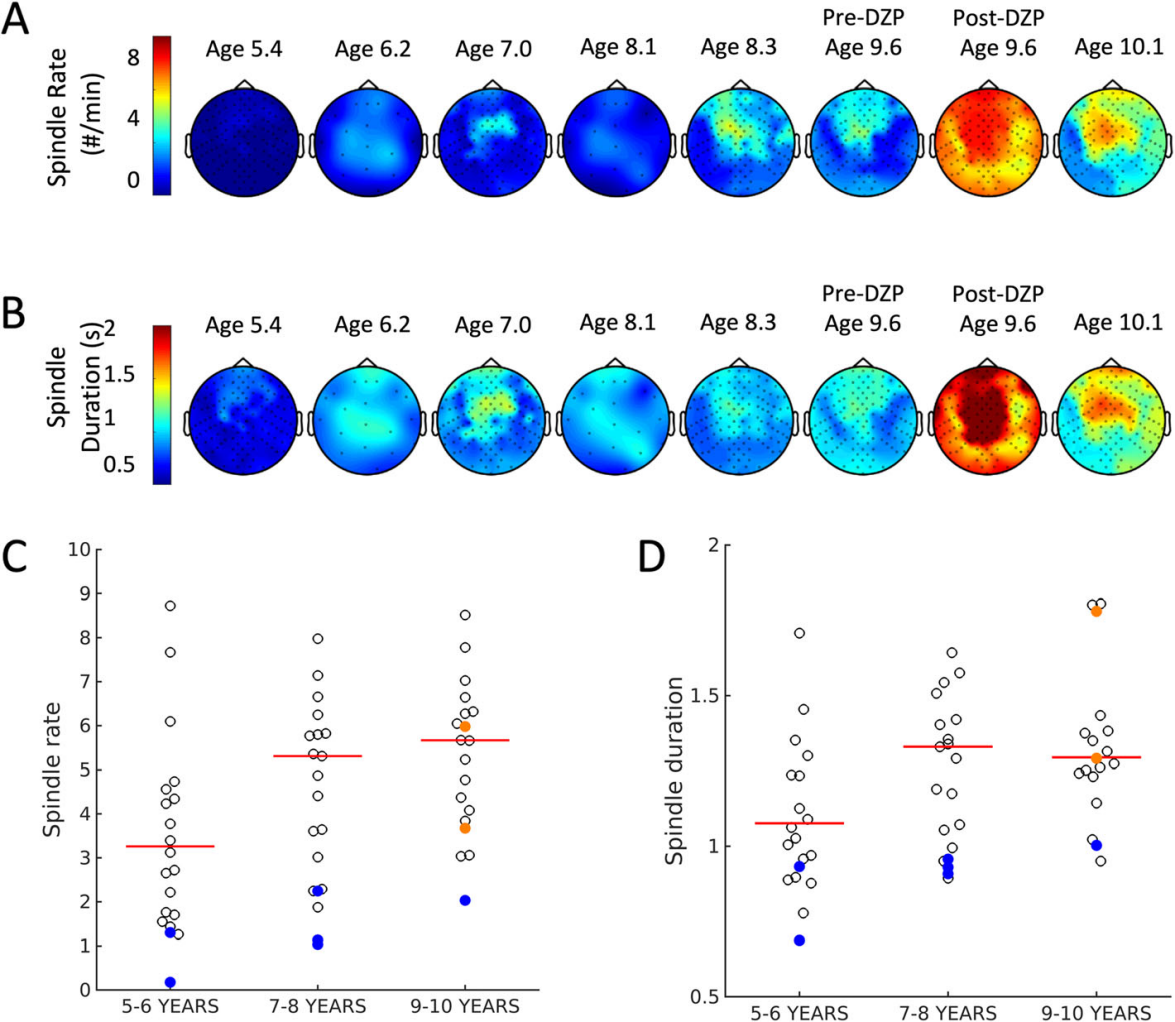
Longitudinal spindle rate and duration during N2 sleep. Topographical map of the **a** spindle rate and **b** duration across ages shows a dramatic increase on the night of high-dose diazepam treatment (red arrow) that is sustained. A transient increase in spindles at ages 6.2–7.0 years coincides with the start of dexmethylphenidate. **c** Spindle rate and **d** Spindle duration for 56 control children using the same detector [32] Open black circles indicate individual children, horizontal red line indicates the median value. The case values are shown in blue (pre-treatment) and orange (post treatment). Here, the higher orange value corresponds to the night of high-dose valium treatment (1 mg/kg) and the lower value to a night several months later on a decreased dose (0.2 mg/kg)

From ages 5.4–9.6 years, the patient’s spindle rate and duration were near-stable (Fig. 5). Mean spindle rate across all electrodes ranged from 0.18 to 2.24 spindles/min during this time and the mean duration across all electrodes ranged from 0.69 to 1.00 s. At age 9.6 there was dramatic overnight increase in spindle rate and duration on the night of high-dose diazepam (1 mg/kg) treatment. Average spindles increased from 2.03 spindles per minute on the night before high-dose diazepam treatment to 5.98 spindles per minute on the night of treatment. Average spindle duration showed a similar increase, from 1.00 to 1.78 s. This increase in spindle rate and duration persisted through the next 4 months while the patient continued treatment with lower doses of diazepam (tapered to 0.2 mg /kg).

Spindle rate typically increases over childhood [31], but absolute spindle values vary by detection techniques. For comparison here, we computed spindle rates from a cohort of age-matched healthy controls (cohort details can be found in [32]) using the same detector (5–6 year olds: *n* = 21; 7–8 year olds: *n* = 19; 9–10 year olds: *n* = 16; Fig. 5c, d). The spindle rate in our patient prior to treatment was below the median rate in the control children (5–6 year olds, median 2.10 spindles / min, range 0.4–7.6; 7–8 year olds: median 3.4 spindles / min, range 0.7–6.1) and after treatment was above the median rate (9–10 year olds, median 3.9 spindles / min, range 1.9–7.3). We note that there was a transient increase in spindle rate between ages 6.2–7.0 years. This temporally coincided with the start of dexmethylphenidate 15 mg at age 6.4 years.

#### Impact of diazepam on power spectrum

Benzodiazepines increase beta activity (12.5–30 Hz) in the EEG [33]. To evaluate the frequencies affected by high-dose valium and whether these overlapped with the sigma band (10–15 Hz), which corresponds to spindle frequency, we computed the power spectra of approximately 5 h of N2 sleep data (290 min for day 1 and 307 min for day 2) prior to and after high-dose diazepam treatment using a 1-s sliding window with 50 percent overlap. The baseline mean was subtracted from each window. A Hanning window taper was applied and the Fast Fourier Transform was calculated. The power spectra of 30 min of artifact-free wake data before and after high-dose diazepam treatment was analyzed in the same manner.

**Fig. 6:**
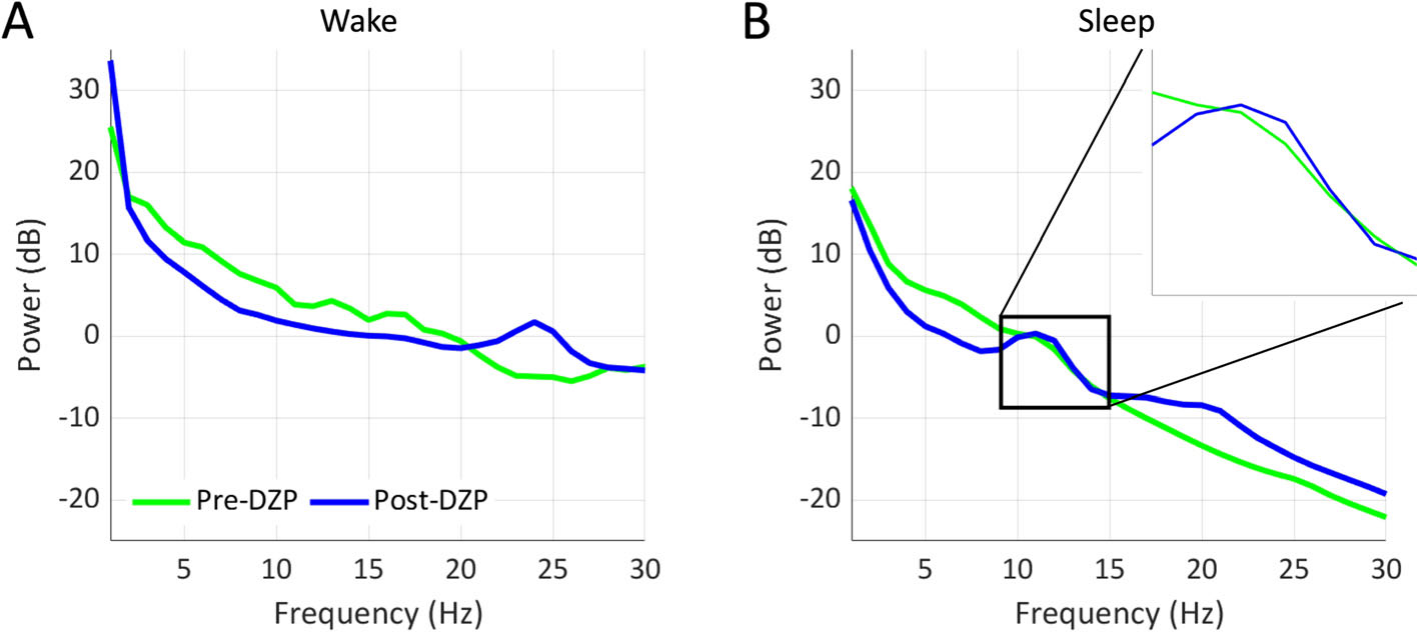
Power Spectral Density curves before and after treatment with high dose diazepam in wake (**a**) and N2 sleep (**b**). During post-treatment sleep there is a discrete bump in the sleep spindle range (10-15 Hz) that is much smaller in pre-treatment sleep. (Inset) magnification of the sleep spindle frequency band. Both post-treatment wake and sleep EEGs show the expected increase in beta band frequencies (~ 20-25 Hz) after treatment with a benzodiazepine

Following diazepam treatment there was a generalized increase in beta activity during wake and sleep as expected from a benzodiazepine (Fig. 6). During wakefulness, there was a distinct increase in 20–27 Hz beta power immediately following high-dose diazepam treatment, compared to prior to treatment. Similarly, during N2 sleep, there was an increase in > 15 Hz beta power following high-does diazepam treatment. In addition, following high-dose diazepam treatment, there was a second discrete increase in 10–15 Hz power during N2 sleep that overlapped with the sigma bump present prior to high-dose diazepam treatment. These findings are consistent with the increase in discrete spindle activity detected using the automated detector.

#### Interictal spike activity and spindle activity

The IIS burden during N2 sleep was quantified using an automated detector, Persyst13 (Persyst Development Corporation, San Diego). This detector has been validated and shown to perform on par with human readers [34].

**Fig. 7:**
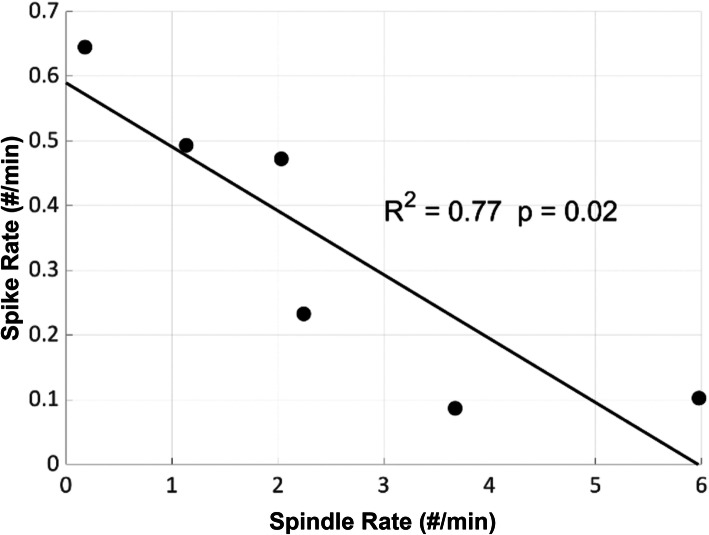
Relationship between detected spike rate and spindle rate. Data obtained using HDEEG recordings are shown here for consistency

IIS activity followed a waxing and waning course over the 5 years of follow up (mean 0.34, range 0.09–0.65). At age 9.6 years, she was found to have an average spike rate of 0.47 spikes/min, with a marked reduction in activity after high-dose diazepam treatment to 0.1 spikes/min. Using a linear regression model, spike rate from the automated detection was inversely correlated to spindle rate over the course the patient’s disease (*p* = 0.02, β − 0.10, 95% CI [− 0.17, − 0.02], *R*^2^ = .77, Fig. 7). Thus, for each increase of 1 spindle per minute, there was a decrease in spikes by 0.1 per minute. This finding is consistent with an antagonistically shared circuitry between IISs and spindles, consistent with observations in CECTS (see Fig. 5 in [27]).

## Discussion

We report longitudinal results in a child with epileptic encephalopathy where treatment with diazepam led to an improvement in cognitive symptoms and an increase in sleep spindles. This case introduces sleep spindles as a proposed electrophysiological biomarker of cognition in epileptic encephalopathy that directly reflects thalamocortical circuit function. A reliable biomarker for the cognitive symptoms in this disease would aid in accurate diagnosis and guide management in this challenging disease.

The current definition of epileptic encephalopathy assumes that epileptic activity interferes with cognitive function [1].The diagnosis of CSWS in particular requires the presence of both electrical features of abundant sleep activated spikes and clinical features of cognitive dysfunction [2]. We propose that cognitive dysfunction in epileptic encephalopathies may not be due to the presence of epileptic spikes per se, but to the absence or disruption of essential graphoelements required for normal cognitive functioning, such as sleep spindles. Our observations here are consistent with prior work looking at sigma-band activity and sleep homeostasis in children with Landau-Kleffner syndrome and Rolandic epilepsy [27, 35–39]. In contrast, sleep-activated spikes are estimated to be present in 3–8.5% of healthy children [40, 41] and do not provide a reliable biomarker of encephalopathy. While clinicians have traditionally relied on trending IIS burden over time, efforts to link IIS and cognitive function in epileptic encephalopathies have not identified a consistent relationship [16, 42–45]. Cognitive deficits can range from subtle to profound, and determining the relationship to an epileptic process rests on clinical judgment. Detection of focal or subtle cognitive deficits may require detailed neuropsychological evaluation [46, 47], which is expensive, time consuming, and not widely available. Repeat neuropsychological testing may be required to identify individual plateaus or regressions. These repeat tests can be complicated by practice effects and may not be reimbursed by health insurance. Furthermore, the cognitive course is not necessarily yoked to seizure course, where developmental concerns may precede seizure onset or persist after seizure resolution [48]. We were fortunate to have overcome many of these obstacles in diagnosis for our case with highly trained neuropsychologists available and a clear regression of skills across time prior to diazepam therapy. Despite these resources, confident diagnosis of CSWS with encephalopathy required 4.7 years in this case. A quantitative biomarker for cognitive dysfunction would enable both early identification and accurate longitudinal surveillance of patients at risk. As sleep spindles are present as early as 6 weeks of age [49]. Disruption of these graphoelements could both indicate pathology and directly affect cognition even in the early onset developmental epileptic encephalopathies.

A growing body of literature points to dysfunction of thalamocortical circuits in epileptic encephalopathies. Sleep spindles are a prominent electrophysiological signal normally generated in the thalamus and propagated by thalamocortical circuits in healthy NREM sleep. Spindles are generated in the thalamic reticular nucleus, which is comprised entirely of GABAergic neurons [9, 50–53]. These neurons project primarily to glutamatergic thalamocortical neurons, which entrain cortical areas to their sigma-frequency rhythms [21]. Corticothalamic neurons send glutamatergic inputs back to the thalamus, producing a feedback loop regulated primarily by GABAergic and glutamatergic neurotransmission [54]. Given the important role that spindles have for learning and memory consolidation [17–19] their disruption could provide a mechanistic biomarker for cognitive dysfunction in epileptic encephalopathies [55].

In vitro, animal and human studies have demonstrated that spikes can be generated by pathologically hijacking the same thalamocortical circuit that generates spindles [21, 22, 30]. Spikes and spindles can be both recorded from single thalamic cells and thalamic slice preparations after different pharmacologic treatments [23, 56, 57]. In humans, thalamic and cortical activity measured with depth electrodes are phase locked during both spindles and spikes [58, 59]. Discrete thalamocortical assemblages generate focal spindles [59] and emerging evidence demonstrates that these focal circuits can also be used to promote focal abnormalities, as in Rolandic epilepsy [60, 61]. The inverse correlation observed here and in prior studies [27] between spikes and spindles supports the competitive relationship proposed between spikes and spindles arising from dysfunction of shared thalamocortical circuitry.

Many children with epileptic encephalopathies have persistent cognitive impairment and there are no proven treatments for this disease [61]. This may be because current treatments target reducing spikes, rather than augmenting normal physiology. The emerging evidence for spindles as a biomarker for cognitive dysfunction opens up transformative opportunities for treatments targeting these rhythms to more directly ameliorate cognitive deficits in epileptic encephalopathies. Currently available pharmacologic [62, 63] and non-pharmacologic [64–67] interventions that increase spindle activity can result in improved sleep dependent memory consolidation. Similarly, interventions that decrease sigma power (e.g. spindle frequencies) impair memory [54]. The thalamocortical circuits that generate sleep spindles involve both GABAergic and glutamatergic channels. High-dose treatment with diazepam, as used in our case, targets GABAergic channels, and therefore may directly support spindle production. Non-pharmacologic interventions to support spindles are also available. Quiet auditory stimuli timed to slow oscillations during NREM sleep increase spindles and memory in healthy controls and have been shown to be safe and tolerated in children with sleep-activated epileptiform spikes [68]. Similarly, selective transcranial direct current stimulation can increase spindle activity and improve memory [69–74]. In our patient, pharmacologic therapy yielded a boost in spindle rate and a concordant improvement in neurocognitive testing. These combined observations suggest that future efforts should invest in defining the relationship between sleep spindles and cognitive deficits in epilepsy and testing available treatment options to mitigate these symptoms.

We observed a modest, transient increase in spindles in our patient that coincided with initiating dexmethylphenidate stimulant treatment for ADHD. Dexmethylphenidate is a stereoisomer preparation of methylphenidate, which indirectly increases catecholamine neurotransmission by inhibiting the dopamine transporter and norepinephrine transporter. Whether stimulants can directly increase spindle rate is unclear, though improved learning as a consequence of improved attention with stimulant treatment may result in increased spindle rate [75].

## Conclusion

We found a dramatic increase in sleep spindles that coincided with cognitive improvement following high-dose diazepam treatment in a child with an epileptic encephalopathy. Sleep spindle deficits provide evidence of thalamocortical circuit dysfunction. Further, sleep spindles are well-established in neuroscience literature as critical for memory consolidation during NREM sleep and offer a direct, established, mechanistic biomarker for the cognitive deficits observed in childhood epileptic encephalopathies. As sleep spindles can be modulated with pharmacologic and noninvasive neuromodulatory approaches, this work identifies a potential novel biomarker and mechanism for cognitive dysfunction and new opportunities for research and treatment of cognitive deficits in epileptic encephalopathies.

## Data Availability

The datasets used and/or analyzed during the current study are available from the corresponding author on reasonable request.

## Abbreviations

IIS: Interictal spikes
NREM: Non-Rapid Eye Movement
CECTS: Childhood Epilepsy with Centro-Temporal Spikes
CSWS: Continuous spike and wave of sleep
WISC: Wechsler Intelligence Scale for Children
WIAT: Wechsler Individual Achievement Test
WRAML-2: Wide Range Assessment of Memory and Learning- Second Edition
